# Supplementary material for: A Critical View of the Application of the APEX Software (Aqueous Photochemistry of Environmentally-Occurring Xenobiotics) to Predict Photoreaction Kinetics in Surface Freshwaters
Source: Molecules. 2019 Dec 18;25(1):9. doi: 10.3390/molecules25010009 (PMC7017383; doi:10.3390/molecules25010009)
Supplement: Supplementary file 1 [file molecules-25-00009-s001.zip › 3.2.4_gcc-4.4.0.zip!/3.2.4_gcc-4.4.0/3.2.4_gcc-4.4.0/doc/octave/html/interpreter/Zeros-Treatment.html]

Zeros Treatment - Untitled


Previous: Example Codes,
Up: Diagonal and Permutation Matrices


---

### 20.5 The Differences in Treatment of Zero Elements

Making diagonal and permutation matrices special matrix objects in their own
right and the consequent usage of smarter algorithms for certain operations
implies, as a side effect, small differences in treating zeros.
The contents of this section applies also to sparse matrices, discussed in
the following chapter.

The IEEE standard defines the result of the expressions `0*Inf` and
`0*NaN` as `NaN`, as it has been generally agreed that this is the
best compromise.
Numerical software dealing with structured and sparse matrices (including
Octave) however, almost always makes a distinction between a "numerical zero"
and an "assumed zero".
A "numerical zero" is a zero value occurring in a place where any floating-point
value could occur. It is normally stored somewhere in memory as an explicit
value.
An "assumed zero", on the contrary, is a zero matrix element implied by the
matrix structure (diagonal, triangular) or a sparsity pattern; its value is
usually not stored explicitly anywhere, but is implied by the underlying
data structure.

The primary distinction is that an assumed zero, when multiplied
by any number, or divided by any nonzero number,
yields \*always\* a zero, even when, e.g., multiplied by `Inf`
or divided by `NaN`.
The reason for this behavior is that the numerical multiplication is not
actually performed anywhere by the underlying algorithm; the result is
just assumed to be zero. Equivalently, one can say that the part of the
computation involving assumed zeros is performed symbolically, not numerically.

This behavior not only facilitates the most straightforward and efficient
implementation of algorithms, but also preserves certain useful invariants,
like:

- scalar \* diagonal matrix is a diagonal matrix- sparse matrix / scalar preserves the sparsity pattern- permutation matrix \* matrix is equivalent to permuting rows

all of these natural mathematical truths would be invalidated by treating
assumed zeros as numerical ones.

Note that certain competing software does not strictly follow this principle
and converts assumed zeros to numerical zeros in certain cases, while not doing
so in other cases. As of today, there are no intentions to mimic such behavior
in Octave.

Examples of effects of assumed zeros vs. numerical zeros:

```
     Inf * eye (3)
     =>
        Inf     0     0
          0   Inf     0
          0     0   Inf
     
     Inf * speye (3)
     =>
     Compressed Column Sparse (rows = 3, cols = 3, nnz = 3 [33%])
     
       (1, 1) -> Inf
       (2, 2) -> Inf
       (3, 3) -> Inf
     
     Inf * full (eye (3))
     =>
        Inf   NaN   NaN
        NaN   Inf   NaN
        NaN   NaN   Inf
```

```
     diag(1:3) * [NaN; 1; 1]
     =>
        NaN
          2
          3
     
     sparse(1:3,1:3,1:3) * [NaN; 1; 1]
     =>
        NaN
          2
          3
     [1,0,0;0,2,0;0,0,3] * [NaN; 1; 1]
     =>
        NaN
        NaN
        NaN
```
